# Beyond the Black Box: Reproductive Strategies of the Black Soldier Fly as a Model for Bridging Evolutionary Biology and Applied Entomology

**DOI:** 10.1111/eva.70249

**Published:** 2026-05-08

**Authors:** Noah B. Lemke, Nalini Puniamoorthy

**Affiliations:** ^1^ KU Leuven Geel Belgium; ^2^ National University of Singapore Singapore

**Keywords:** adult nutrition, behaviour, capital‐income breeding, mass rearing optimisation, mate choice, phenotypic plasticity, speciation, welfare

## Abstract

The black soldier fly (BSF; *Hermetia illucens* ) is rapidly emerging as a model for evolutionary biology and insect biotechnology. Although larval biology has been extensively characterised, the reproductive biology of adults remains comparatively understudied. In this review, we synthesise the most recent empirical work on physiology, behavioural and chemical ecology to open the ‘black box’ of BSF reproduction, focusing on processes that span eclosion to senescence. We highlight pre‐ and post‐mating mechanisms that determine overall reproductive fitness: from mating latency, lekking dynamics, courtship and copulation, to sperm transfer, storage and oviposition. We discuss these processes within the framework of sexual selection theory. Several notable characteristics of BSF reproduction differ from traditional insect models. These include a hybrid capital‐income breeding strategy (adults do not *need* to feed but can benefit from supplemental nutrition), protandry (early male emergence), sex‐specific longevity that varies with mating status and a lek‐like mating system. In addition, females possess morphologically complex sperm‐storage organs, providing ample opportunity for intense post‐copulatory sexual selection. Recent work shows that environmental factors such as light, humidity, temperature, substrate volatiles and rearing design strongly influence reproductive output in industrial settings, highlighting the potential for BSF to bridge fundamental and applied research. We propose a novel conceptual framework that integrates these elements and outline key unresolved questions (e.g., mechanisms of sperm precedence, female control of fertilization, reproductive barriers, drivers of speciation etc.). This interdisciplinary model supports both fundamental insights into the evolution of reproductive traits and provides practical improvements for optimizing industrial mass‐rearing.

## 1 Introduction

In the past decade, the black soldier fly (BSF), *Hermetia illucens* (L.) (Diptera: Stratiomyidae) has attracted growing interest both in scientific research and in industrial applications. As an efficient decomposer of organic waste with a high degree of nutritional flexibility, BSF larvae are widely studied for converting organic waste into protein and fertiliser, making it a keystone species in sustainable agriculture and circular food systems (Tomberlin and van Huis 2020; Tomberlin et al. 2022). At the same time, BSF is increasingly recognised as a model organism for fundamental studies in evolutionary biology and behavioural ecology (Tomberlin et al. 2025), and yet, despite this potential, the adult reproductive biology remains largely underexplored. Industry‐driven research has overwhelmingly focused on larval life history traits, rendering the reproductive biology of the adult as a ‘black box’. A recent bio‐economic analysis even suggested that improving larval growth and conversion efficiency yields higher economic returns than improving reproductive traits. Yet, reproduction is the ‘heartbeat’ of any species' life cycle and, for a mass‐reared insect like BSF, neglecting the reproductive phase limits both scientific understanding and long‐term industrial optimization. Past reviews treated either the fundamental (Lemke et al. 2023) or the applied (Barrett, Chia, et al. 2023; Kortsmit et al. 2023; Meneguz et al. 2023) aspects of BSF biology in isolation. We argue that these perspectives are together complementary, and this review integrates both into a holistic understanding. We acknowledge that a complete view of the holometabolous BSF life cycle is likewise essential (from egg, to larvae, to pupae, to adult), but here, we concentrate on the adult stage and the mechanisms driving reproductive outcomes because this is the area where the most substantial knowledge gaps continue to persist. Moreover, other reviews have a much more specific focus on genetics and selective breeding in farmed insects (Hansen et al. 2025), but here, we can only focus on genetics in brief.

Insects exhibit a remarkable diversity in reproductive strategies across lineages (Wilson 1999) and emerging evidence suggests BSF is unique in many ways. For instance, there are many well‐studied insects that are of relevance to both fundamental and applied research: classical models for developmental and cell biology such as the common fruit fly ( *Drosophila melanogaster* ); vectors of human diseases like mosquitoes (*Anopheles* spp., *Aedes* spp.); pollinators such as the Eurasian honeybee ( *Apis mellifera* ), as well as agricultural pests such as the red flour beetle ( *Tribolium castaneum* ), the pea aphid (*Acrythosiphon pisum*) and the house fly ( *Musca domestica* ), the Olive fly (*Bactrocera olea*); and even other insects recently domesticated for food and feed such as the house cricket ( *Acheta domesticus* ) and the mealworm ( *Tenebrio molitor* ). Interestingly, most of these species are income breeders, which in a strict sense, means that adults must feed to reproduce. By contrast, BSF adults do not *require* feeding for gametogenesis. Instead, successful adult reproduction relies almost entirely on fat body reserves accumulated during the larval stage occur (Tomberlin and Sheppard 2002). Adult foraging can nevertheless extend lifespan and enhance reproductive output (Zhang, Ng, et al. 2025; Kortsmit et al. 2025). This largely capital breeding (but hybrid) strategy has critical evolutionary implications: Because adult nutrition cannot compensate for the nutritional history experienced as larvae, any variation in larval diet is directly reflected in adult reproductive performance, especially in captive or industrial systems where many producers do not feed adults. However, the possibility of feeding as adults presents the possibility of interesting behavioural trade‐offs (Kortsmit et al. 2025) for future research to explore.

Black soldier flies also exhibit protandry. That is, males emerge several days before females (Tomberlin et al. 2002), though the timing of emergence is sensitive to larval nutrition (Zhang, Ng, et al. 2025) and is linked with size dimorphism (Generalovic et al. 2025). The asynchronous emergence of both sexes and of entire cohorts creates complex reproductive dynamics (Lemke, Li, Dickerson, et al. 2025). In both nature and captivity, adults tend to segregate by sex (Tomberlin and Sheppard 2001; Lemke et al. 2024) and form lek‐like swarms (Lemke, Smith, Smink, et al. 2025) enabling a polygynandrous mating system in which multiple individuals of each sex may mate repeatedly (Manas, Venon, et al. 2025). Moreover, industrial studies suggests that reproduction in BSF can be responsive to environmental cues (Chia et al. 2018; Addeo et al. 2022). Beyond the temperature and humidity constraints of a tropical fly (Lemke, Smith, Smink, et al. 2025), factors such as like light spectra and intensity (Zhang et al. 2010), specific substrate volatiles that attract females for oviposition (Zheng et al. 2013) and even the physical design of mating cages (Grosso et al. 2025) can significantly influence mating success and fecundity in captivity. For the most part, however, what is understood about BSF reproductive biology stems from captive populations in laboratories or industries that are typically artificially selected for agricultural waste conversion. Yet, BSF exist worldwide (Kaya et al. 2021), in presumably a wide range of ecological contexts. Recent genomic research on wild‐derived, commercial and selectively‐bred BSF lines confirmed that domestication, prolonged captive rearing and selective breeding rapidly alter population structure, leading to significant genetic differentiation among these groups (Silvaraju et al. 2026). As such, understanding the features of BSF reproduction has broad implications for both evolutionary biology and applied entomology.

For decades, classical model organisms such as *Drosophila melanogaster* Meigen 1830 (Diptera: Drosophilidae) have dominated the fundamental research landscape (Kohler 1994; Markow 2015). Although invaluable, *D. melanogaster* represents a narrow slice of insect diversity, and its utility has often stemmed from laboratory tractability (Ankeny and Leonelli 2011). In fact, laboratory strains of *D. melanogaster* often differ significantly from wild populations, which can limit the generalizability of findings (Kohler 1994; Markow 2015). This over‐reliance on a single system creates practical risks; for instance, recent NIH budget changes have impacted funding for the *D. melanogaster* bioinformatics repository, FlyBase (flybase.org, May 2025), highlighting the urgent need to diversify and develop alternative research models.

The emergence of BSF as a new model is therefore timely. This species occupies a key important phylogenetic transition between aquatic‐ and terrestrially‐developing flies (Lemke et al. 2023), sitting centrally within the Dipteran lineage between many economically important Nematocera (including mosquitoes, gnats, midges, black flies, drain flies, sand flies and crane flies) and Brachycera flies (including hover flies, Tsetse flies, blow flies, bot flies, Tachnid flies, true fruit flies, stalk‐eyed flies and vinegar flies) (Yeates and Wiegmann 2005; Lambkin et al. 2013). As mentioned, its hybrid capital‐income breeding strategy offers a powerful system for studying life‐history trade‐offs in ways not possible with strict income breeders. Furthermore, its lek‐like mating system is similar to more distantly related insects such as Lepidoptera like the tobacco hornworm, *Manduca sexta*, as well as broader comparisons to many other animal groups where this behaviour has convergently evolved (Höglund and Alatalo 1995). Altogether, the wide global distribution, pronounced larval nutritional flexibility, rapid genomic divergence and high industrial relevance of BSF together provide a powerful system for investigating phenotypic plasticity and the mechanisms between selection, genetic drift and reproductive isolation.

Classically, what makes a good model organism is one that has most or all the following traits: short lifespan, rapid reproduction, small size, ease of maintenance and well‐understood genetics (though in reality, often what becomes entrenched in research is that which has gained a certain amount of inertia). Organisms like *D. melanogaster* and *C. elegans* fit this bill and over the last century became entrenched as models for understanding human genetics and development. But now with the advent of high‐throughput ‐omics technology, the concept of model organisms has expanded beyond these pillar organisms, enabling a more holistic focus towards understanding complex ecological and evolutionary phenomena. The perspective has shifted and now a model organism is not just those which have fully characterised genetic and/or cellular architectures, but those which enable the answering of specific questions (Box 1). Specifically, we believe that BSF are an ideal system to address:

- How nutritional legacies acquired during development impact adult fitness and reproductive strategies.
- How light exposure, humidity, temperature and other abiotic conditions structure the dynamics of a mating system.
- How mating systems evolve and shift under the intense pressures of domestication and artificial selection.
- The relative strengths of pre‐ and post‐copulatory sexual selection in a capital breeder.
- The mechanisms by which reproductive barriers form and contribute to isolation.

BOX 1 **What makes BSF a model organism in the classic sense?**

*Short lifespan*: In captivity, BSF take ~5 days to hatch from eggs, ~2–3 weeks to develop through 6 larval instars, ~2 weeks to complete metamorphosis via pupation and ~1 week to reproduce (though this is an oversimplification, as each of these can vary substantially, because the velocity of insect development depends directly on temperature (Ratte 1985)). The generation time is thus generally between 6 and 7 weeks, meaning 7 generations can occur per year, but can be sped up with higher temperatures (Chia et al. 2018) or under artificial selection (García‐Castillo et al. 2025). In the wild, BSF breed year‐round in the tropics (Tomberlin and Sheppard 2001), but this is restricted to fewer generations in temperate regions where presumably they must overwinter (Spranghers et al. 2017).

*Rapid reproduction*: Reproduction is triggered in mature adults after exposure to UV‐AB and blue‐green spectra light. Females carry between 300 and 900 eggs, which evolved as a strategy typical among ‘R‐selected species’ that compensate for developmental instability and high morbidity with high fecundity. But with reasonable fertility and survivorship in captivity, this leads to rapid population growth in excess of what is needed to maintain a breeding colony, such that typically 10% of eggs are used to replenish the breeding stock, with the remainder being devoted to production.

*Small size*: As insects, BSF are relatively small, allowing them to be reared and maintained in a small space. Larvae can reach 250 g each, and the largest adults are between 18 and 20 mm (but the smallest can be ~5 mm). However, this makes BSF relatively large compared to other insects (considering most insect diversity is housed within dark taxa which are generally tiny/miniscule Diptera and Hymenoptera (Chimeno et al. 2022)). Coupled with their dark bodies, this enables BSF to be more easily studied via videography and computer vision applications (Nawoya et al. 2024; Smetana et al. 2025) because less specialised hardware is needed to capture high contrast images of adult behaviour.

*Ease of cultivability*: Trays of larvae are relatively easy to maintain on shelves in rearing chambers (to some extent, they are ‘plug‐and‐go’ to start but require monitoring). Mating can be achieved in dimensions as small as 30 × 30 × 30 cm (Nakamura et al. 2016). However, raising BSF is not entirely without its difficulties. Throughout their development, BSF larvae produce high amounts of heat (Li et al. 2023) and gaseous waste products (e.g., carbon dioxide (CO_2_), ammonia (NH_3_), etc.) (Coudron et al. 2024) as they digest their substrate, which must all be properly ventilated (Lalander et al. 2020). Additionally, flying adults need to be maintained in enclosures (compared to sessile or non‐flying species). Moreover, as a tropical species, optimal conditions include high humidity (~70%–80% RH) (Cammack and Tomberlin 2023), temperature (~30°C–35°C) (Chia et al. 2018) and intense lighting (8000 μW/cm^2^ of UV‐AB light) that each mirror wild optima (Lemke, Smith, Smink, et al. 2025), and environmental controls can be costly to maintain at scale.

*Characterised genetics*: BSF have 7 chromosomes with a genome size of approximately 1.01 Gb with roughly 67% of this comprised of repetitive, noncoding, or transposable elements (Zhan et al. 2020). Recent work has begun to characterise BSF genetics (i.e., via a chromosome assembly (Generalovic et al. 2021), parentage assemblies (Hoffmann et al. 2021; Dufresne et al. 2025), quantification of inbreeding in captivity (Rhode et al. 2020) and more). Moreover, a CRISPR‐Cas9 systems have been developed (Zhan et al. 2020; Sui et al. 2024), making gene editing possible (though the soft chorion makes CRISPR injections into BSF eggs difficult) and low‐cost imputation methods for low coverage whole genome sequencing have recently been developed for BSF (Muchina et al. 2026).

**FIGURE 1:**
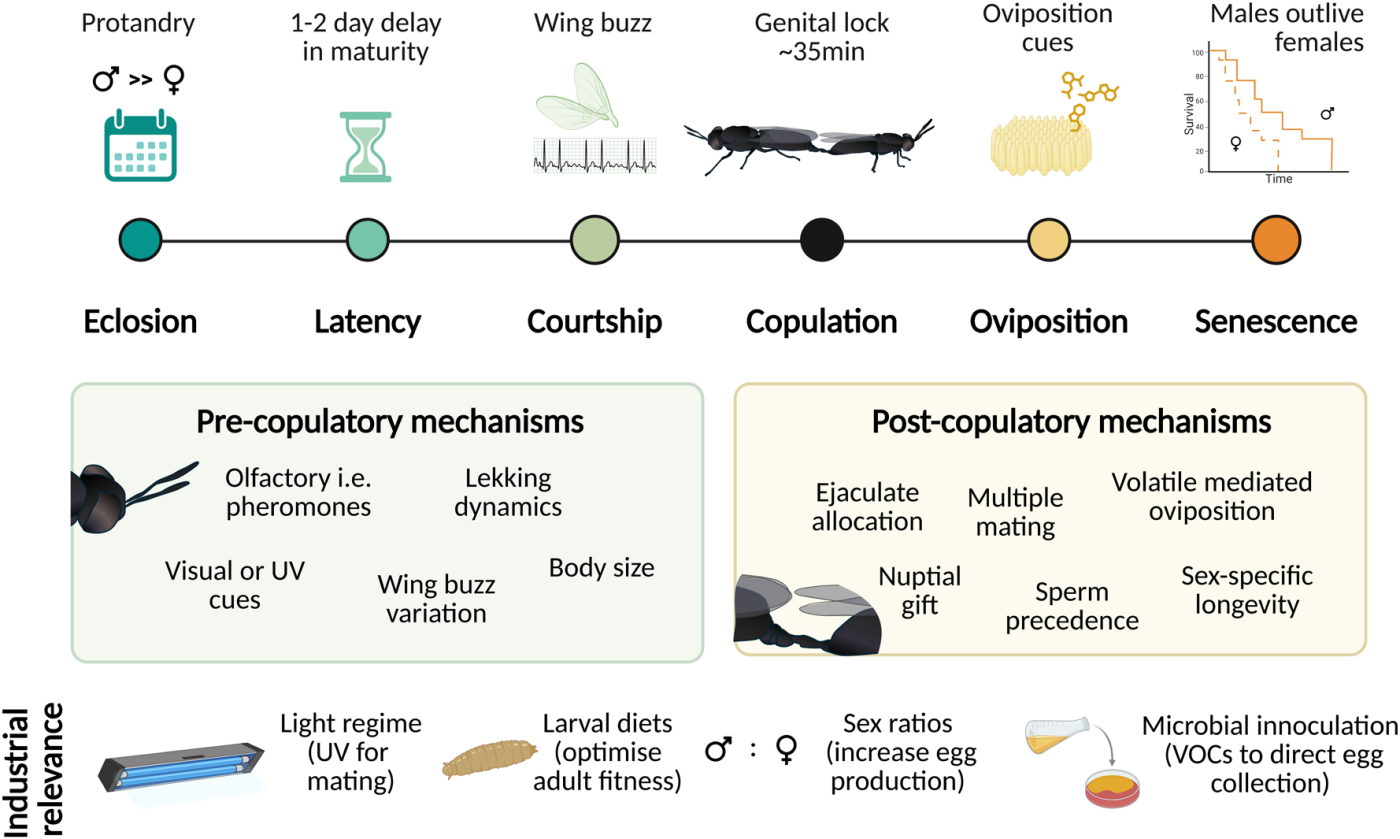
Graphical overview.

To address these questions, we synthesise emerging empirical evidence on BSF reproduction into a comprehensive conceptual framework (Figure 1), identifying key research gaps. We examine processes that govern reproductive outcomes from adult emergence to senescence, including sexual maturation, courtship, copulation, sperm dynamics, as well as oviposition. By placing these elements within the framework of sexual selection theory, we aim to reposition BSF as a powerful and versatile model for modern evolutionary biology and behavioural ecology research. We also discuss how external factors, from lighting regimes to substrate cues, can influence reproduction and emphasise opportunities to translate fundamental knowledge into applied rearing practices.

## 2 Mechanisms and Dynamics of Reproduction

Reproduction in BSF is a complex process shaped by nutritional history, social context and environmental cues. Recent work suggests that from sexual maturation to senescence, both males and females exhibit dynamic physiological and behavioural adaptations that challenge earlier assumptions of BSF as a monogamous or behaviourally uniform species (Nakamura et al. 2016; Park et al. 2017; Giunti et al. 2018; Freitas Spindola 2019; Macavei et al. 2020; Malawey et al. 2020; Surendra et al. 2020; Awal et al. 2022). To address these complexities, we examine how the pre‐copulatory, copulatory and post‐copulatory phases determine overall reproductive success.

### 2.1 Pre‐Copulatory Phase

#### 2.1.1 Latency and Sexual Maturation

Protandry is a defining feature of BSF, with males emerging several days before females (Tomberlin et al. 2002; Meyermans et al. 2025). This strategy is common in butterflies and other insects, occurring in ~36% of investigated insect species (Teder et al. 2021). It is thought to allow males to monopolise access by being present and ready to mate as soon as females become receptive, thereby minimizing the time females spend unmated (Zhang, Henawy, et al. 2025; Fagerström and Wiklund 1982). The duration of this developmental gap between male and female emergence, also known as sexual bimaturism (SBM), is modulated by larval nutrition and interacts with body size and longevity (Generalovic et al. 2025); larvae that take longer to develop become larger adults. As the size difference between females and males increases, so does the degree of SBM (Teder et al. 2021; Kortsmit et al. 2025). This reproductive timing is intrinsically linked to the BSF's life history where adults do not *need* to feed or forage prior to mating (Tomberlin et al. 2002). Instead, they rely almost exclusively on the resource accumulation during the larval stage (Lemke et al. 2023; Harjoko et al. 2023). However, BSF are not strict capital breeders and supplemental nutrition can benefit adult fitness (Thinn and Kainoh 2022; Klüber et al. 2023; Barrett et al. 2025), though this is not always consistently observed (Lemke et al. 2023; Coudron et al. 2025). Recent work even suggests a potential trade‐off, where providing adults with supplemental nutrition may reduce their investment in mating behaviours (e.g., slowing their time‐to‐first‐mating, decreasing wing‐buzzing duration, mating durations and mating frequency) (Kortsmit et al. 2025). Lastly, another recent study has uncovered 23 gustatory receptors, 3 of which correspond to sugar receptors that have analogues in *Drosophila* (Merle et al. 2026). Neurobehavioural assays confirm that BSF can indeed taste and respond to sugar, with females responding more strongly than males (Merle et al. 2026). Although BSF have a reduced set of sugar receptors compared to other flies, they have nonetheless retained them throughout millions of years of evolutionary history. Their apparent preference for complex sugars against a white background (Romano et al. 2020), suggesting they are pollinators of white, nectar‐producing flowers (Bertinetti et al. 2019). But despite any love of sugar the adults might possess, larval nutritional legacy remains the primary driver of BSF reproductive potential (Lemke et al. 2023; Harjoko et al. 2023). Although provisioning adults with water intuitively aids welfare by preventing dehydration (Sheppard et al. 2002), reproduction in BSF can proceed without adult feeding, a key distinction from most income‐breeding insect models.

##### 2.1.1.1 Male Maturation and Sperm Dynamics

Upon eclosion, male BSF possess some sperm (ca. 3000–11,000 spermatazoa) (Munsch‐Masset et al. 2023) but they typically delay mating for 1–2 days. This latency likely serves to synchronise their reproductive readiness with female oocyte maturation (Tomberlin et al. 2002; Meyermans et al. 2025) and allows them time to establish positions within lekking aggregations (Lemke, Li, Dickerson, et al. 2025). Indeed, in lek‐based systems such as in true fruit flies (Tephritidae) where males display in groups, reproductive success is often determined by position within the lek, e.g., a central position or on specific trees (Shelly 2018).

Male BSF continue to produce sperm throughout life, but testes size shrinks with age suggesting a lifetime sperm production limit of approximately 50,000 spermatozoa per male (Munsch‐Masset et al. 2023; Manas et al. 2024). Recent experimental work suggests that this finite sperm resource can be managed strategically. In response to perceived sperm competition risk from other males, BSF males can adjust their sperm investment, indicating an adaptive allocation of ejaculates (Manas, Labrousse, and Bressac 2025), which has likewise been shown to occur in other insects (Shuker and Simmons 2014). Sperm length may also play a role in competitive fertilization success. BSF have relatively long sperm (Malawey et al. 2019, 2020), being 6 times longer than the average length of other animals with internal fertilization (Munsch‐Masset et al. 2023), but several *Drosophila* are outliers possessing exceptionally long sperm (Lüpold et al. 2016). Although inadequate larval nutrition in BSF can generate smaller males with shorter sperm (Zhang, Henawy, et al. 2025), it is still unclear if there are potential trade‐offs between quantity versus quality of sperm production, nor whether these translate to direct fitness effects.

##### 2.1.1.2 Female Maturation and Fecundity

In contrast to males, females emerge with immature ovaries and undergo synovogenic egg development post‐emergence, with synchronous egg maturation from a single ovariole (Munsch‐Masset et al. 2023). This distinguishes BSF from many other proovigenic insects whose eggs are all mature at the onset of their adult life, as well as many insects whose fecundity is linked to both the number and variation of ovarioles (Moore 2014). This strategy in BSF is a likely adaptation to stochastic nutritional environments that BSF larvae often inhabit (i.e., in heterogenous wastes). In fact, rather than being predetermined, female fecundity in BSF is instead tightly correlated with adult body size (Spearman's *ρ* = 0.73) which depends on larval diet (Gobbi et al. 2013; Shrestha et al. 2025). This direct link between larval nutrition, adult size and egg number leads to significant natural variation in both fecundity (number of eggs) and fertility (hatching success) (Laursen et al. 2024; Zhang and Puniamoorthy 2025). Consequently, when evaluating reproductive output, it becomes challenging to disentangle what proportion of this variability in *fertility* is female‐ mediated (e.g., egg viability) versus male‐mediated (e.g., sperm quantity and quality), as both are influenced by their shared larval developmental history.

#### 2.1.2 Navigation and Lekking

A central challenge for reproducing insects is locating a mate, leading to the emergence of diverse strategies like pursuing, patrolling, hilltopping, perching and ambushing, just to name a few (Alcock 1984, 1987). To date, the exact mechanisms by which BSF locate mating sites in the wild remain largely unknown (Lemke et al. 2023). Observations of captive populations suggest that flies may be guided by gradients in light because newly eclosed adults from darkened pupation areas (Ferdousi and Sultana 2021) will be attracted towards illuminated zones (Dortmans et al. 2017; Coudron et al. 2025). This is potentially exploited in industrial mass‐rearing systems for automated counting because flies can be funneled through a pinhole (James et al. 2024). Moreover, this behaviour may mirror a natural pattern because BSF larvae are negatively phototropic and often seek dark crevices prior to pupation; the subsequent positive phototaxis of adults would guide them out of emergence sites and into open, illuminated areas where mating can occur (Giannetti et al. 2022).

Although natural lekking sites have been described as occurring hundreds to potentially thousands of meters from emergence zones (Tomberlin and Sheppard 2001; Lemke et al. 2023), it is unclear whether stochasticity (e.g., updrafts of wind) or specific cues drive BSF navigation to leks in the wild. Because females are readily attracted to rotting substrates, field observations are often biased towards these ovipositing females near anthropogenic wastes. However, mating adults are conspicuously absent from these same locations. Indeed, a recent study in Costa Rica failed to find any mating BSF within a radius of 30 m, despite the prevalence of female flies within 7.5 m of an oviposition site (Lemke, Smith, Smink, et al. 2025). A spatial segregation between the larval media/oviposition sites with mating sites is a key feature of BSF reproductive ecology (along with that of other lekking species) and implies that females must take significant flights on their limited energy reserves to both mate and lay eggs. Such could explain the sex‐specific differences in adult longevity (Tomberlin et al. 2002; Harjoko et al. 2023; Zhang, Ng, et al. 2025), as well as increased female receptibility for sugars (Merle et al. 2026) and the possibility of male nuptial gifts (Harjoko et al. 2023) that could together be supplemental nutrition for BSF on these flights.

Unlike other Diptera that follow sex pheromones to locate conspecifics (Wicker‐Thomas 2007) or other chemical trails or orient towards specific landmarks, BSF might not emit any long‐range attraction pheromones (Lemke et al. 2023), making their ability to form dense swarms particularly enigmatic; though this still needs to be confirmed experimentally. Within the artificial environment, mating flies often display spatial segregation away from the oviposition sites and towards less‐humid microenvironments (unpublished data). This suggests that in an industrial setting, dedicated mating arenas and oviposition areas within the cage could enhance reproductive success (Salari and De Goede 2024) (Refer to Box 2 and Section 4.3 for further discussion).

BOX 2 **Debates on mate discrimination in BSF.**

A central question in BSF reproductive biology is whether adults, particularly males, can discriminate between sexes and assess mate quality. For instance, BSF exhibit sexual dimorphism in wing interference patterns created by differences in structural colouration (Butterworth et al. 2021; Rebora et al. 2024). These honest signals putatively are detected visually thanks to high photoreceptive sensitivity of the ommatidia in BSF compound eyes (Oonincx et al. 2016). Evidence suggests that visual neural pathways are more developed in males than females (Barrett, Godfrey, et al. 2022); but this then only adds to the conundrum of why males might mount other males (Giunti et al. 2018). Recent work in BSF suggests that acoustic signals may play a role in sex discrimination once mounted, where the wing‐fanning (=buzzing) duration is much longer between male–male mating attempts than either female–male mating attempts or successes (Kortsmit et al. 2025). Moreover, because wing‐fanning duration is not influenced by larval diet (Kortsmit et al. 2025), this may be a canalised mechanism underlying mate discrimination.

However, others contend that BSF cannot discriminate between sexes or kin at all (Giunti et al. 2018; Laudani et al. 2024). Reports of male–male mating interactions led to the hypothesis that BSF males are largely indiscriminate (Julita et al. 2020; Jones and Tomberlin 2021; Chiabotto et al. 2024; Kortsmit et al. 2025). Supporting evidence for this hypothesis comes from the lack of sexual dimorphism in their cuticular hydrocarbon (CHC) profiles (Lemke et al. 2023), which typically facilitate communication during physical contact (Ingleby 2015). For instance, the CHCs of *Drosophila* serve as short‐range signals indicating sex, reproductive status, age and social rank, which together mediate attractiveness and can be manipulated to dupe rivals and mates (Laturney and Billeter 2016; Holze et al. 2021). However, the New World Screwworm Fly *Cochliomyia hominivorax* is a counter‐example which, like BSF, lacks sexual dimorphic CHCs altogether (Pomonis 1989).

Interestingly, the examination of same‐sex sexual (SSS) behaviours in 111 other insects and arthropods revealed that SSS is more common between males than females and is highly associated with captivity, high‐rearing densities and the presence of female pheromones (Scharf and Martin 2013). This suggests that for BSF, SSS behaviours are largely an artifact of their captivity and of laboratory studies using small enclosures, e.g., as small as 30 × 30 × 30 cm (Nakamura et al. 2016). Still, it is unclear as to how BSF differentiate amongst individuals of different species, sexes, ages and conditions; how decisions are made in response to the integration of long‐ and short‐range signals; nor whether high population densities are linked to SSS behaviours.

#### 2.1.3 Courtship and Mounting

Courtship is typically initiated by female flight or entry into a swarm. In captivity, females have been observed to momentarily leave their perch (on artificial plants or walls) to enter male swarms occurring near artificial light sources (Lemke et al. 2024) before either returning to perch or descending *in copula*. This observed behaviour appears to mirror natural history descriptions in which females visit the lek where males are already present (Tomberlin and Sheppard 2001; Lemke et al. 2023). The timing of these interactions is structured, with male flight activity peaking in the late morning and early afternoon, followed by a peak in female activity later in the day (Lemke et al. 2024). Such temporal patterns in perching and flight activity mirror that of other diurnal lekking insects (Michiels and Dhondt 1989) (Refer to Box 3).

Courtship in BSF involves the multimodal integration of visual, acoustic, vibratory and (presumably) olfactory and gustatory signals (Wicker‐Thomas 2007). Males perform aerial displays of wing‐fanning, which combined with wing‐interference patterns (WIPs) putatively serve as visual signals for conspecifics (Rebora et al. 2024). These visual displays are accompanied by vibratory ‘songs’ produced by wing‐buzzing (Giunti et al. 2018; Laudani et al. 2024; Kortsmit et al. 2025), similar to many other groups of insects (e.g., crickets grasshoppers) (Baker et al. 2019). BSF might alter the harmonics of their buzzing by rapidly modulating the frequency of their wingbeats, as is the case in *Aedes aegypti* (Diptera: Culicidae) (Aldersley and Cator 2019). Besides pitch and tone, mating ‘songs’ might need to be performed with the proper rhythm (Eberhard and Gelhaus 2009) and duration to be accepted by prospective mates. And although mating songs encode signals about prospective mates, the significance of these being used to predict mating success in BSF comes with conflicting results. Some experiments suggest a positive correlation between duration of wing buzzing and mating success (Giunti et al. 2018), others have reporting the opposite trend (Laudani et al. 2024) or no correlation at all (Kortsmit et al. 2025).

When males mount females, they typically attempt genital engagement from behind (Julita et al. 2020). If a female is receptive, successful pairs ‘lock’ their flexible genitalia (Rollinson et al. 2025), allowing them to rotate their position into a stable, tail‐to‐tail posture that is maintained during copulation while perched on a surface. Although some remain mounted from behind (Chiabotto et al. 2024), the ability to rotate between the two positions may allow the pair to take flight and relocate in response to disturbance (pers. obs.).

BOX 3 **Debates on lekking in BSF.**

The mating system of BSF is described as ‘lekking’ (Tomberlin and Sheppard 2001) or ‘lek‐like’ (Birrell 2018), in which males aggregate at spatially distinct sites which females visit strictly to mate (Alcock 1990). Lekking is a complex phenomenon, characterised by several behaviours including a lack of male parental care, sex‐based dispersion, territoriality and the absence of resource monopolization. However, lekking itself is no longer viewed within a strict definition (Alcock 1987). Each of these criteria can be considered as an independent continuum in multidimensional parameter space similar to the Hutchinsonian niche (Hutchinson 1957), enabling a more holistic view of lekking systems. Moreover, the lek mating system itself also can be modelled along a gradient of increasing resource dependence and mate monopolization (Emlen and Oring 1977; Parker 1978; Thornhill and Alcock 1983) and can shift dynamically if these underlying factors change. For instance, under high population densities and when males are unable to control access to females, this leads to scramble competition polygyny (Herberstein et al. 2017). Conversely, when females become rare, the mating system can shift to either resource‐defence polygyny or female‐defence polygyny once males start to monopolise female resources or the females themselves (Buzatto and Machado 2008), respectively. Mating systems that fall shy of these two extremes, but still have some similarity to a true lek, are called ‘lek‐like’. This behaviour has been described in one other congener, the Agave soldier fly, *Hermetia comstockii* (Diptera: Stratiomyidae) (Alcock 1990). However, more distantly related soldier flies do not lek: *Merosargus cingulatus*, which mate near their oviposition site of decomposing vegetation (Barbosa 2011), and *Inopus rubiceps* (Macquart) (Diptera: Stratiomyidae) which engages in scramble competition (Alcock 1990).

Some field and lab observations document stable male aggregations (Tomberlin and Sheppard 2001; Lemke et al. 2024), whilst other reports mention no aggregation except within ephemeral mating balls involving multiple males (Permana et al. 2020), although these might be incorrectly mistaken for leks. Overall, there exists debate as to whether lekking as a whole is preserved in captivity (Lemke et al. 2023) and whether large aggregations are a prerequisite for mating (Laudani et al. 2024). Although some studies report a clear behavioural segregation of the sexes (Lemke et al. 2024), other aspects of lekking are often not reported because most detailed behavioural studies in BSF are typically done at small scale, because manual observations of BSF behaviour become impossible even at modest densities. High densities of flies are sometimes thought of as being a prerequisite to successful mating (although when looking across the tree of life, this is only the case for obligate social breeders). Instead, it follows that BSF should still be able to mate in the absence of competitors and indeed mating in lone pairs of BSF has recently been described (Jensen et al. 2025). However, when BSF are reared at the industrial scale, i.e., in cages housing up to 20,000 or more individuals per cubic meter, new evolutionary optima may be favoured, whereby alternative mating strategies, such as males forming satellite leks or engaging in opportunistic scramble encounters become more successful, selecting for a new mating system over successive generation. However, to date there exists a lack of conclusive evidence of the spatiotemporal hierarchy within BSF leks, whether this is directly linked with variation in reproductive fitness and whether environmental context can cause shifts in the rules structuring such a mating system.

### 2.2 Copulatory Phase

#### 2.2.1 Copulatory Mechanics, Duration and Sperm Transfer

Copulation in BSF is marked by genital engagement lasting approximately 33 min (Manas, Labrousse, and Bressac 2025), the onset of which is defined by a physical ‘lock’ between the male and female genitalia. This is made possible due to the morphology of the male terminalia (i.e., the lateral extensions of the gonostylus) that bend medially inwards, effectively functioning like a claw and allowing a male to grasp the female's terminalia (Rollinson et al. 2025). This can be contrasted with a genital lock that might be formed via the swelling of genitalia, which famously occurs in *Canids* (Carnivora: Canidae) (Beach 1970). Once the lock commences, rival males apparently cease interruption attempts. Moreover the duration of the genital lock appears mostly unaffected by adult size but varies with age (Manas, Labrousse, and Bressac 2025; Manas, Venon, et al. 2025), suggesting that BSF might not engage in mate guarding, which in other species can arise as a mating tactic to prevent females from mating with rivals.

Initial matings might last between 32.5 ± 13.4 (mean ± SD) minutes, though research suggests mating duration generally increases with each successive mating up to 50.2 ± 26.6 min for the fifth mating (Manas, Venon, et al. 2025). Additionally, overall mating duration is prolonged in sibling crosses (Laudani et al. 2024) and amongst individuals that experienced poor larval nutrition (Zhang, Ng, et al. 2025). Crucially, sperm transfer is not a continuous process. Dissection of females at different times throughout mating suggests that sperm is delivered only in the lattermost portion of copulation (e.g., after ~25 min), following the transmission of seminal fluids (Manas, Labrousse, and Bressac 2025). Copulation then ceases when both sexes disengage, after which both sexes perform grooming behaviours, with females typically grooming for a longer period than males (~300 versus 100 s) (Giunti et al. 2018; Laudani et al. 2024) (Box 4).

BOX 4 **Reproductive skews in BSF.**

Mating success in BSF is far from evenly distributed. It can be dependent on both biotic—e.g., sex ratio, density (Hoc et al. 2019)—and abiotic factors (e.g. light, temperature, stressors (Dearlove et al. 2025)) and experimental studies suggest that mating frequencies can vary dramatically across populations and colonies (Jones and Tomberlin 2021; Dickerson et al. 2024; Lemke et al. 2024; Lemke, Li, Dickerson, et al. 2025; Meyermans et al. 2025). In fact, in equal sex ratio populations of 500 male and 500 female BSF, only 43–120 mating events were observed over a week, indicating that a minority of males likely secure the bulk of matings (Meyermans et al. 2025). In much smaller groups of 15 male and 15 female BSF, nearly half of the males never mated. Half of the successful males mated repeatedly, producing a pronounced reproductive skew (Manas, Venon, et al. 2025). This pattern suggests population substructures (driven via non‐random mating) and a low effective population size, which are hallmarks of lek‐type mating systems in which a single or a few dominant males monopolise fertilizations. Thus far, no studies have addressed how genetic diversity might be maintained over time for BSF in light of the lekking paradox (Kotiaho et al. 2001). It is also unclear if relative reproductive fitness varies as a function of mating rates in BSF (*sensu* Bateman's gradients (Bateman 1946)), though some work showed consanguineous breeding in captivity can eventually lead to low effective population sizes (Rhode et al. 2020).

#### 2.2.2 Sperm Storage

After copulation, BSF females store sperm in multiple spermathecae, enabling fertilization of several egg clutches over time. Females possess complex sperm storage organs: three spherical spermathecae, attached to three sclerotised rods that end in hinges, which are connected to three separate fishnet canals (that contain sperm) (Munsch‐Masset et al. 2023; Bruno et al. 2025). The total number of stored sperm can range from a few hundred to several thousand (Manas et al. 2024) and it may take about 48 h for approximately 50% of transferred sperm to reach the sperm storage reservoirs. Recent work demonstrated that a single ejaculate is sufficient to fill a female's sperm reservoirs in excess, which challenges the previous hypothesis that females were sperm limited (Permana et al. 2020). Instead, a single mating can support multiple egg clutches. In addition, over time, the quantity of stored sperm declines but sperm viability remains relatively stable, suggesting that females may digest surplus sperm because BSF females do not dump sperm (Manas et al. 2024). In addition, the social environment or presence of rival males appears to influence sperm storage because females that mate in the presence of conspecifics also retained more sperm than those isolated post‐copulation (Manas, Labrousse, and Bressac 2025) (Box 5).

BOX 5 **Re‐mating in BSF.**

For BSF in captivity, the majority of matings occur on the first or second day after being introduced to mating cages, but continue to occur at a low rate after this, reaching a plateau between days 4 and 6 (Tomberlin et al. 2002; Dickerson et al. 2024; Lemke et al. 2024). Behavioural assays have observed male BSF to mate up to four (Chiabotto et al. 2024; Laudani et al. 2024), five (Jensen et al. 2025) or six times (Manas, Venon, et al. 2025) and females up to twice (Chiabotto et al. 2024) but higher female polyandry, up to five times, has been supported by parentage assignments (Hoffmann et al. 2021). Importantly, recent work has shown this temporal pattern appears to generally be consistent across experiments and are highly correlated (98%) to oviposition events 2–3 days later (Lemke, Li, and De Smet 2025). These findings highlight the potential for strong postcopulatory sexual selection that influence ejaculate‐female interactions and overall reproductive fitness in BSF.

### 2.3 Post‐Copulatory Phase

#### 2.3.1 Oviposition

Following copulation, female BSF typically begin laying eggs between 4 and 6 days of their introduction into the breeding environment, though the pre‐oviposition period can be greatly lengthened at temperatures below 30°C or shortened with increasing temperatures up to BSF's maximum threshold (Chia et al. 2018). This timing varies based on biological age (Dickerson et al. 2024) and mating experience (Permana et al. 2020). Females lay eggs in small, dry crevices near the actual oviposition substrate that is often wet and humid (Sheppard et al. 2002). Indeed, adaptions of eggs to thrive in dryer substrates (e.g., development of chorion respiratory structures (Wigglesworth and Beament 1960), and protective mucilaginous secretions around egg clutches) allowed ancestral Brachycera to transition away from fully aquatic development into amphibious life and eventually fully terrestrial niches (Lemke et al. 2023) by protecting against egg desiccation (Peñalver et al. 2022). The timing of oviposition itself can be both genetically and environmentally influenced. For instance, a study investigating the effect of full‐sibling crosses on reproduction showed that inbred lines exhibit longer pre‐oviposition intervals than their outbreeding controls (Laudani et al. 2024), whereas adult feeding shortens the time between copulation and oviposition (Barrett et al. 2025), but as discussed may have the effect of increasing time to first‐copulation and decreasing mating duration (Kortsmit et al. 2025).

Female fecundity, i.e., the number of eggs laid, can vary as a function of adult nutrition (Thinn and Kainoh 2022; Klüber et al. 2023; Barrett et al. 2025), body size (Gobbi et al. 2013), age (Dickerson et al. 2024) and even genetic relatedness (Laudani et al. 2024). Several recent studies suggest female BSF can oviposit multiple times (Jones and Tomberlin 2021; Hoffmann et al. 2021; Chiabotto et al. 2024; Laudani et al. 2024), though others argue BSF oviposit only once (Sibonje 2024). Originally these additional clutches were thought to be completely infertile (Nakamura et al. 2016) but female BSF can lay two to three fertile clutches after even a single mating (Manas et al. 2024). Examination of egg clutches collected from breeding cage experiments has revealed that both fertility and neonate viability can vary in substantial ways, with a portion of eggs (from multiple clutches) not developing at all, a portion developing eyespots but otherwise not hatching, a portion hatching but not surviving, as well as a portion that hatches, survives and successfully reproduces (A. Dickerson, unpublished data); although environmental factors (e.g., heat, desiccation), could likewise contribute to variation in offspring viability.

Likewise, delaying mating (e.g., as an artifact of experimental set‐up) is suspected to cause females to haphazardly lay eggs when females lay single (or additional) clutches (Dickerson et al. 2024; Muraro et al. 2024; Lemke et al. 2024) far away from the larval substrate, as can environmental stress (Lemke, Li, Dickerson, et al. 2025). Off‐target egg laying can quickly snowball because females are attracted to VOCs released by eggs (Klüber et al. 2024), which might then signal to additional females to lay their eggs in off‐target locations. Fecundity and fertility (i.e., the proportion of larvae that hatch) can trade‐off at industrial scale (Hoc et al. 2019) and recent study suggests that larval nutrition can induce phenotypic plasticity in both female fecundity as well as fertility (Zhang and Puniamoorthy 2025) (see Box 6).

BOX 6 **Dynamics of oviposition site selection.**

In nature, mating and oviposition often occur at spatially distant and distinct sites (Tomberlin and Sheppard 2001; Lemke et al. 2023, 2024), which one hypothesis states could have evolved to dilute predation pressure (Rathore et al. 2023). BSF larvae are polyphagous and are able to thrive on a wide range of substrates. Most accounts of wild‐trapped females are typically associated with anthropogenic waste (Sripontan et al. 2017; Nyakeri et al. 2017; Ewusie et al. 2019; Ferdousi et al. 2022; Purkayastha and Sarkar 2023; Sable and Chavan 2024; Yaseen et al. 2025). Controlled experiments have revealed that BSF prefer to oviposit (depending on the choices available to them) in grain/vegetable/fruit wastes (Kotzé and Tomberlin 2020; Laksanawimol et al. 2023; Zim et al. 2023) as well as the medium near aged carrion (Kotzé and Tomberlin 2020) and manure (Zim et al. 2023). However, experiments suggest that female BSF do not necessarily select the oviposition site which coincides with highest larval fitness (Boafo et al. 2023; Tekaat 2024), indicating there are other ecological factors at play as well as maternal‐offspring conflicts.

The selection of oviposition sites (i.e., =larval foraging sites) is undoubtably guided by complex interkingdom olfactory cues emitted by microbial communities as well as conspecifics (Zheng et al. 2013; Klüber et al. 2024; Thomas et al. 2024; Zhang, Henawy, et al. 2025; Klammsteiner et al. 2025). Key attractants that could increase oviposition and which have so far been identified include tetradecanoic acid, sulcatone and acetophone. BSF olfactory responses to these have been confirmed by amputating BSF antennae for choice test experiments (Klüber et al. 2024). Importantly, there could be a physiological ‘switch’ that is triggered post‐mating for females (Lemke, Li, Dickerson, et al. 2025), as has been found in tephritids (Jang et al. 1999), because unmated/virgin female BSF showed no clear behavioural preferences for these chemical cues, whilst gravid females exhibited a clear response to the tetradecanoic acid (Klüber et al. 2024), or various plant‐based attractants (Laksanawimol et al. 2023). This potentially contradicts a hypothesis in which attractant timing should be delayed until after mating to reduce behavioural trade‐offs/conflicts in decision making (Lemke, Li, and De Smet 2025; Lemke, Li, Dickerson, et al. 2025); and moreover, points to the possibility that the significant results generated in this study are more causally linked to differences in substrate maturity across treatments (and the differences in volatiles they released), rather than synergies between adult ontogeny, female olfaction and the breeding cycle. It has also been theorised that BSF males may not be as responsive to these volatiles, especially because female antennae have longer flagellum than males, which would suggest increased function (Pezzi et al. 2021). However, electroantennographic recordings confirm the olfactory abilities of BSF of both sexes in response to various volatiles with substantial overlap in their responses (Piersanti et al. 2024), suggesting that differences in behavioural responses of each sex to volatiles must then be driven by sex‐specific genetic/neural architecture, activations of neural switches, or tunings via hormonal modulation. Moreover, it is unclear whether seminal fluid proteins modulate such a switch in females; molecules similar to sex peptide in *Drosophila* that are transferred in the male ejaculate, are famous for triggering a cascade of behavioural and physiological changes in females (Chapman et al. 2003). Interestingly, they evolved as a way to increase male fitness often at a cost to female fitness, i.e., via a sexual conflict (Wigby and Chapman 2005). Overall, disentangling these drivers across the many levels of biological organization is key to both understanding BSF reproductive evolution, i.e., (i) the changes over time in reproductive traits and systems across lineages, and (ii) the accumulation and evolution of reproductive barriers that can reduce gene flow and underlie population genomic structure, as well as optimizing mass‐rearing protocols.

#### 2.3.2 Aging and Senescence

Aging in adult BSF is often marked by visible changes in the physical condition of flies including wing damage, limb loss and desiccation. These likely occur from repeated attempts to mate or oviposit despite declining energy reserves, as well as inadequate humidity and/or water resources in the breeding environment. In other mass‐reared Diptera, injuries are often associated with harassment (Meza et al. 2025) and in captive male tephritids typically become hyper‐aggressive under artificial selection (Briceño et al. 1999). Observations from both laboratory and industrial settings report older BSF frequently crashing to the cage floor during flight or even spinning erratically after having broken one of their wings, which can explain some of their injuries. In BSF, because such physical changes are quite conspicuous, aging can be estimated based on the decreasing opacity of the abdominal ‘windows’ (Harjoko et al. 2023), through which one can observe changes in the stored nutrient reserves (which also include changes in colour). These translucent windows are thought to have evolved to mimic the petiole of *Polistes* paper wasps (James 1935; Alcock 1990). Observing colour changes in the abdominal windows can serve as a non‐invasive indicator of biological aging and has been successfully adopted for population management in industrial settings (Salari and De Goede 2024).

Longevity in both sexes can be extended by provisioning adults with water (Tomberlin and Sheppard 2002) and food (Lemke et al. 2023; Barrett et al. 2025). Interestingly, the seminal components transferred by males to females may contribute to female longevity, potentially acting as nuptial gifts (Harjoko et al. 2023) for females to digest (Manas et al. 2024) by supplying amino acids and/or lipids that may support egg development and/or metabolic function. Older males exhibit reduced mating frequency (Dickerson et al. 2024) and fertility due to declining sperm viability (Malawey et al. 2020). This means that housing mixed ages of BSF adults can reduce the efficiency of reproduction at the industrial scale (Lemke, Li, Dickerson, et al. 2025). For instance, older, less competitive males may interfere with females mating with younger mates, as observed in tephritid flies (Papanastasiou et al. 2011), which in these species has necessitated habitat design that features distinct areas for each sex (Meza et al. 2025). Similarly, female clutch size and egg viability decline with age and egg collections from mixed age cohorts will undoubtedly exhibit a greater variability in survival and overall fitness.

## 3 Implications for Evolutionary Biology

The black soldier fly ( *Hermetia illucens* ) combines a suite of life‐history traits that make it a promising model for understanding the evolution of reproductive systems. As mentioned, being a (hybrid) capital breeder (Stephens et al. 2009), adult reproduction relies almost entirely on larval‐derived reserves, thereby decoupling gametogenesis and courtship investment from adult foraging. This reshapes sexual selection dynamics relative to other income‐breeding insects (Stephens et al. 2009) and anautogenous blood‐feeding Diptera (which require a blood meal to produce eggs), creating potential trade‐offs among larval resource allocation, somatic growth, longevity and reproductive output (Miller 2005). Multiple mating by females influences sperm competition among males, a ubiquitous force in insect reproduction (Parker 1970). Comparative studies show how sperm traits evolve under such competition: *Drosophila bifurca* produces the longest sperm in the animal kingdom, whereas lepidopterans package sperm into nutrient‐rich spermatophores (Pitnick et al. 1995; Vahed 1998). In BSF, recent studies document polygynandry, steep reproductive skews, extended sperm storage and possible cryptic female choice, including ejaculate digestion and selective sperm retention (Muraro et al. 2024; Manas et al. 2024; Manas, Labrousse, and Bressac 2025). These findings provide a framework to quantify pre‐ vs. post‐copulatory sexual selection gradients (and their relative strengths to one another) under varying sex ratios, densities and age structures, especially because lek‐like aggregations can be experimentally manipulated in captivity by altering population demography (Jones and Tomberlin 2021; Dickerson et al. 2024; Lemke, Li, Dickerson, et al. 2025), the physical structure of the breeding environment (Lemke et al. 2024; Grosso et al. 2025) and abiotic conditions.

Phenotypic plasticity (West‐Eberhard 1989) driven by larval diet influences key reproductive traits in BSF, including adult body size, fecundity and sperm length, that also interacts with heritable variation to shape evolutionary potential (Gobbi et al. 2013; Shrestha et al. 2025; Zhang and Puniamoorthy 2025). Intentional selection on larval feed conversion rates, adult body weights and fecundity can yield rapid responses but increase the risk of inbreeding depression (Meyermans et al. 2025), especially as a consequence of unintentional artificial selection (e.g., for BSF that perform well in captivity, such as those that survive abiotic shocks, those that oviposit in traps and those that produce eggs with increased desiccation resistance, etc.). Laboratory studies show BSF can adapt within generations to suboptimal diets (Jiggins 2024), supporting their use in experimental evolution. Recent genomic surveys indicate substantial diversity among global BSF populations (Ståhls et al. 2020), including rapid genomic differentiation between strains under artificial selection (Silvaraju et al. 2026). This raises the possibility of BSF actually being a cryptic species complex (Ståhls et al. 2020; Generalovic et al. 2023; Athanassiou et al. 2024) and provides an avenue to examine potential mechanisms governing reproductive isolation under domestication. Besides the obvious isolation of these strains from one another in time and space, reproductive barriers may be driven by local adaptations to different feedstocks, rearing conditions and selection regimes that produce phenotypic differences (e.g., microbiota, morphology, behaviour). The interaction between industrial selection pressures and ancestral reproductive behaviours such as the separation of mating sites from oviposition sites (Tomberlin and Sheppard 2001) enables research to test of how evolutionary ‘holdovers’ persist or decay in closed systems (Lemke et al. 2024) by comparing patterns in captivity to those in the wild (Lemke, Smith, Smink, et al. 2025).

### 3.1 Cryptic Female Choice, Sperm Competition and Sexual Conflict

Cryptic female choice occurs when females, after mating with males, utilise an internal mechanism to bias which sperm fertilise her eggs. In insects, dozens of such mechanisms have been uncovered (Shuker and Simmons 2014). For instance, insects such as *Drosophila*, crickets and butterflies can bias sperm use through differential storage or ejection of sperm from less‐preferred males (Eberhard 1991). The highly specialised sperm storage organs of BSF females are capable of retaining more sperm than required to fertilise a single clutch (Manas et al. 2024). Retention duration, sperm viability and selective sperm use suggest potential for cryptic female choice as described in other stratiomyids (Barbosa 2009, 2011, 2012, 2015). Females may regulate sperm uptake or storage or bias fertilization toward preferred males, but such mechanisms remain largely untested in BSF.

Likewise, males can differentially adjust their sperm transfer, which is often called cryptic *male* choice (Shuker and Simmons 2014). This often occurs in response to perceived sperm competition risk (Manas, Labrousse, and Bressac 2025). Moreover, as mentioned, sperm length in BSF (Malawey et al. 2019, 2020) is unusually long for internally fertilizing animals and is sensitive to larval diet quality (Zhang and Puniamoorthy 2025), suggesting potential trade‐offs between sperm quality and quantity that could shape outcomes among sperm competing within the genital tract of the female and her sperm storage organs. However, the degree to which sperm precedence follows ‘last male wins’ patterns, as seen in other Diptera, is unknown, nor how this pattern may subsequently break down with high amounts of remating (Zeh and Zeh 1997; Laturney et al. 2018) or as flies senesce (Mack et al. 2003). Indeed, this phenomenon is quite complex. Last‐male sperm precedence may arise—not just males remove or displace their rivals' sperm—but if the female herself digests/ejects sperm from her genital tract prior to a subsequent mating (Luck et al. 2007; Schnakenberg et al. 2012). Determining the balance of these processes in BSF will require targeted paternity analyses across sequential matings.

The mating system of BSF may be subject to sexual antagonism or sexual conflict (Bedhomme et al. 2009; Candolin 2019; Plesnar‐Bielak and Łukasiewicz 2021) because interactions between males and females can impose fitness costs on one sex while benefiting the other, potentially driving coevolutionary dynamics between the prevalence of male manipulation and female resistance (Chapman and Partridge 1996). Often, the number of matings that optimizes fitness for males is higher than it is for females (Wigby and Chapman 2005), driving such conflicts as additional matings for females may be less advantageous for their fitness, but obtaining too few matings will be sub‐optimal for male fitness. BSF males can mate up to 4–5 times (Jensen et al. 2025), whereas females might only remate twice (Chiabotto et al. 2024). Male harassment to secure these extra matings may interfere with female oviposition or even induce injury (Pitnick and García–González 2002), such that increased exposure to males may be negatively associated with female lifetime fitness (Fowler and Partridge 1989; Morrow and Gage 2001). In addition, males may transfer seminal fluid proteins (e.g., sex peptides) that influence female re‐mating latency or stimulate reproductive development (Kubli 1992). These act to increase male fitness at the cost of female fitness (Wigby and Chapman 2005). But conversely, mating in BSF has been shown to reduce male longevity whilst increasing females', suggesting a potential trade‐off between mating frequency and fitness for each sex (Harjoko et al. 2023).

Together, these processes place BSF among the minority of capital‐breeding insects (e.g., Lepidoptera of the families Notodontidae, Arctiidae, Lymantriidae, Saturniidae and Lasiocampidae (Tammaru and Haukioja 1996) and others) in which both sperm competition and cryptic female choice can be studied under experimental conditions by manipulating larval diets or nutritional availability. The combination of tractable rearing, measurable reproductive traits and manipulable social context makes BSF a suitable system for testing predictions of post‐copulatory sexual selection theory, from sperm allocation models to sexual conflict evolution.

### 3.2 Reproductive Barriers and Potential Drivers of Speciation

A recent population genetic study of wild and industrial BSF strains revealed extensive rapid differentiation that can occur under domestication (Silvaraju et al. 2026). Using genome‐wide restriction‐site‐associated DNA sequencing, the study identified clear population structure, reduced heterozygosity in long‐term domesticated lines and signatures of selection across eleven BSF populations (including one long‐term domesticated line, five selectively bred lines, three wild‐derived populations and two commercial strains). These results echo earlier work using mitochondrial DNA, indicating that *cytochrome oxidase subunit I* (*COI*) haplotype diversity is high enough to possibly warrant considering BSF a species complex. BSF strains have divergences of 4.3%–4.9% (Ståhls et al. 2020), which far exceeds the standard 2%–3% for a ‘barcode gap’ (Hebert et al. 2003). Indeed, BSF specimens from around the globe can sometimes look morphologically distinct to a discerning eye (pers. Obs.). Together, the mitochondrial and nuclear evidence suggest that BSF may consist of multiple genetically distinct lineages that are on independent evolutionary trajectories.

The genomic data from Silvaraju et al. (2026) further reveal that selective breeding and prolonged captive rearing can rapidly erode genetic diversity and shrink effective population size, potentially accelerating the emergence of reproductive barriers such as mechanical isolation or reduced hybrid fitness. An earlier study noted that mating experiments between Singapore wild‐caught flies and Spanish commercial strains (*COI* distance ~3.7%–4.0%) produced successful hybrids, indicating that reproductive incompatibilities were not yet absolute between those two strains. However, many facilities that now maintain multiple strains from across the globe (e.g., in KU Leuven, National University of Singapore) have anecdotal reports indicating that crossing some (but not all) of these strains is challenging (unpublished data).

Early work proposed the potential for mechanical/structural isolation, because large body‐size differences among BSF might cause a mismatch in genitalia size and thus create a barrier to reproduction especially under extreme directional selection. However, a recent study shows that male BSF genitalia are hypo‐allometric (Rollinson et al. 2025), allowing males to mate with females across a wide range of body sizes and increase their pool of available mates (Eberhard et al. 1998), and thus is unlikely to be a physical barrier to reproduction. Indeed BSF of multiple size‐classes successfully mate, although with dramatically increased variability (Jones and Tomberlin 2021). Nevertheless, other pre‐zygotic variables, including strain‐specific microbiomes (Silvaraju et al. 2024) or even behavioural variation in visual and acoustic courtship signals, have yet to be explored as barriers to gene flow. Post‐zygotic barriers (hybrid inviability, hybrid infertility and hybrid breakdown) have not been explicitly tested, but the observed genomic divergence and reduced heterozygosity in domesticated lines raise the possibility that hybrids could suffer reduced fitness, especially under the intense selection regimes typical of industrial rearing (Silvaraju et al. 2026).

## 4 Implications for Industrial Applications

Industrial BSF production depends on predictable, high‐output reproduction, yet adult reproductive biology often remains a major bottleneck, especially when it comes to maintaining genetic diversity and performance over time. Although larval traits dominate economic models (Zaalberg et al. 2024), adult reproduction is the rate‐limiting step in closed‐cycle rearing (Boller 1972), leading some industry producers to specialise strictly in BSF egg/neonate production and selling these to other producers who then can specialise in waste conversion (though for others their operations are centralised around their own breeding programme). Optimizing adult phase management requires translating evolutionary and behavioural insights into practical protocols.

### 4.1 Genetics and Population Structure

High genetic diversity is present among global BSF strains (Ståhls et al. 2020; Sandrock et al. 2021; Kaya et al. 2021; Generalovic et al. 2023), but recent work demonstrates that domesticated and selectively bred lines can exhibit markedly reduced heterozygosity and form distinct population clusters, highlighting the genetic consequences of intensive breeding programmes (Silvaraju et al. 2026). Globally, 66% of allelic diversity resides within Neotropics strains, although industrial strains often harbour a narrowed gene pool due to breeding stocks having shared provenance, founding events from colony establishments and repeated genetic bottlenecks (Ståhls et al. 2020; Sandrock et al. 2021; Kaya et al. 2021). The detection of reduced heterozygosity in captive colonies (Rhode et al. 2020; Generalovic 2023; Silvaraju et al. 2026) underlines the need for controlled breeding strategies that preserve adaptive capacity. However, the precise tolerance of BSF to inbreeding and the optimal outcrossing regime remain unresolved (Fowles and Nansen 2019). For instance, although some studies document inbreeding depression and reduced progeny in captive populations (Rhode et al. 2020; Laudani et al. 2024), another shows the opposite, with increased production traits as a result of inbreeding (Cai et al. 2022).

In addition, we encourage industrial consideration for correlated (or antagonistic) trade‐offs between improved larval traits and adult traits (behaviour, postcopulatory selection, etc.), given the effects of larval nutritional legacy on downstream reproductive outcomes. Continuous breeding cycles (whereby new flies are introduced into cages prior to the removal/death of old flies) lead to the interactions (or lack thereof) between BSF of mixed ages, sizes, conditions and reproductive statuses (whereas in the wild, morbidity effects may cause post‐reproductive flies to quickly die). Although continuous production is initially less‐labour intensive, it has the significant drawbacks of making it difficult to precisely identify and control for variation that accrues throughout various steps in the industrial production cycle, as well as perpetuating this variation because any collected eggs will be fertilized and laid by a heterogenous group of flies (Lemke, Li, Dickerson, et al. 2025). Recent work suggests that within captive populations, size‐class (Jones and Tomberlin 2021; Julita et al. 2025), age (Dickerson et al. 2024; Lemke, Li, Dickerson, et al. 2025), mating status/experience (Permana et al. 2020) and genetic relatedness/homogeneity (Laudani et al. 2024; Meyermans et al. 2025) each influence mating rates, sperm competition intensity and egg viability. Size differences increase variation in such outcomes (Jones and Tomberlin 2021) and overrepresentation of old or non‐competitive males appears to depress productivity (Dickerson et al. 2024; Lemke, Li, Dickerson, et al. 2025), thus managing the instantaneous operational sex ratios (iOSR) by rotating discrete breeding cohorts via batches is predicted to reduce skewed mating success and increase effective population size (in addition to managing the larval conditions which give rise to adult variation in the first place such as controlling for variation around the mean of larval parameters).

### 4.2 Nutritional Management

Adult BSF's access to water and nutrient supplements (e.g., honey, pollen, or sugar water) can extend lifespan, shorten pre‐oviposition intervals and increase egg production under several conditions (Thinn and Kainoh 2022; Klüber et al. 2023; Barrett et al. 2025). Although classically described as capital breeders, providing BSF adults supplemental nutrition pushes them slightly towards the middle of the capital‐income continuum (Davis et al. 2016). Interestingly, in Hymenoptera and Lepidoptera income breeding is negatively associated with ovigeny index (i.e., the initial egg load divided by the potential lifetime fecundity) but allows for individuals to engage in mixed strategies to compensate for deficient nutrition acquired as larvae. However, adult nutrition is variable too and has negative consequences for income breeders (Jones and Widemo 2005). The opposite is true as well, with capital breeding and strict proovigeny (i.e., the emergence of a female's entire potential lifetime complement of eggs) being associated with stable and predictable larval conditions (Pélisson et al. 2012). For BSF, because larval diet can influence adult reproductive strategies (Kortsmit et al. 2025), supplementing the nutrition of adult breeding stocks may be necessary to sustain high fecundity, especially to compensate for the fact that waste remediation efforts by industry will necessarily require larvae to be fed on abundant, but low quality feed stocks. One common argument against providing food to adults is that it will make cages difficult to clean; however, at scale, BSF production is far from pristine, as the metabolic wastes of BSF, eggs laid off‐target and dead flies all accrue on cage surfaces (pers. obs.); however, the benefits of adult feeding must be weighed against potential drawbacks such as increasing longevity (especially in continuous production cycles) and operational costs (Lemke et al. 2023).

### 4.3 Sex Ratios and Habitat Design

Lek‐like behaviours that persist among captive BSF (Tomberlin and Sheppard 2001; Lemke et al. 2023, 2024) suggest that spatial separation of the mating and oviposition zones within cages, proper provisioning of microclimates and a precise control of light gradients can enhance reproductive success (Lemke, Li, Dickerson, et al. 2025). There are similar efforts in mass‐reared fruit flies (Diptera: Tephritidae), which require specialised habitat design with separate environments for male and female premating development (Liedo et al. 2007; Meza et al. 2025), though as mentioned, this is because industrial rearing invertedly selects for male aggression (Briceño et al. 1999). A recent report indicates that BSF microhabitat preference is related to their age, so different cohorts of flies may be physically isolated through cage compartmentalization to (Salari and De Goede 2024) which ultimately should help to maintain a proper iOSR and age‐structure of the breeding zone, increasing fertile egg production. In addition, cage designs should consider enhanced structural complexity by including artificial or live plants (Meneguz et al. 2023). For BSF, the increased perching area provided by the plants has been shown to increase mating rates, although the magnitude of the effects varies with the density of the plants provided (Lemke et al. 2024; Grosso et al. 2025). Theoretically there should be an optimum: too little is not beneficial, but too much may be a physical or visual hinderance to mating (Lemke et al. 2024). Habitat design for captive animals is important to consider, because not only does it form the basis of animal welfare (by shaping the animals physical and social experiences), proper design should interact with BSF biology to not only promote natural behaviours but also increase fitness and fertile egg production (Barrett, Chia, et al. 2023). For instance, it has been speculated that BSF will vertically (or spatially) stratify based on underlying biology (i.e., dominant, young, or large males occupying a central or higher/lower position); because the same is true for other insects and lekking species (Rathore et al. 2023). Providing perches may allow BSF (and especially females) to conserve energy and help to reduce any negative effects of crowding (Lemke et al. 2024). Although not necessary for BSF, live plants can help modulate ambient humidity via transpiration of water vapor, as well as to ameliorate air quality by assimilating ammonia (NH3) into their leaf tissues (Zayed et al. 2023). By mimicking natural conditions, the effects of providing plants can potentially reduce stress, as is the case in captive cockroaches (Blattodea) (Free and Wolfensohn 2023), though in practice it may be logistically unfeasible for BSF producers to cultivate/purchase and maintain live plants as well.

### 4.4 Chemical Ecology and Oviposition Control

The interplay between VOC‐mediated oviposition cues and deterrents opens opportunities for chemical manipulation of BSF oviposition behaviour in industrial mass‐rearing. In specific, the BSF symbiotic microbiota community undergoes successive shifts throughout its ontogeny (Klammsteiner et al. 2026). It is these microbes (i.e., colonizing the surface of eggs and larvae, as well as living in the gut and substrates of the larval media) (Heussler et al. 2023) which release VOCs that have evolved to be attractive to mature females, driving detection and oviposition behaviour (Zheng et al. 2013). But not all VOCs are attractive to BSF; rather, some compounds (e.g., decanoic acid) have been shown to delay oviposition behaviour in BSF, whereas others increase off target laying. Because synthetic blends combining multiple VOCs elicit stronger responses than individual components alone (Thomas et al. 2024), this means that a precise blend can be engineered as part of a push–pull strategy (Menger et al. 2015; Cui et al. 2022) to more precisely direct egg‐laying both away from cage materials and towards the trap. Once realised, this will improve collection efficiency, reduce off‐target oviposition and facilitate egg harvesting.

In addition to directing oviposition, aromatherapy using both plant essential oils (e.g., α‐copaene derived from sweet orange, grapefruit, guava, papaya and mango; methyl eugenol; raspberry keytone; α‐Pinene; Zingerone) (Zeni et al. 2021) and synthetic compounds (e.g., Trimedlure) (Shelly et al. 1993) has been shown to artificially stimulate lek formation and mating in many tephrids. Of course, because tephritids have co‐opted secondary plant metabolites as a rendezvous‐signal for their mating aggregations (often in fruit trees), a similar effect of a secondary plant metabolite on BSF is mere speculation until such a plant‐insect interaction can be uncovered.

## 5 Conceptual Model

**FIGURE 2:**
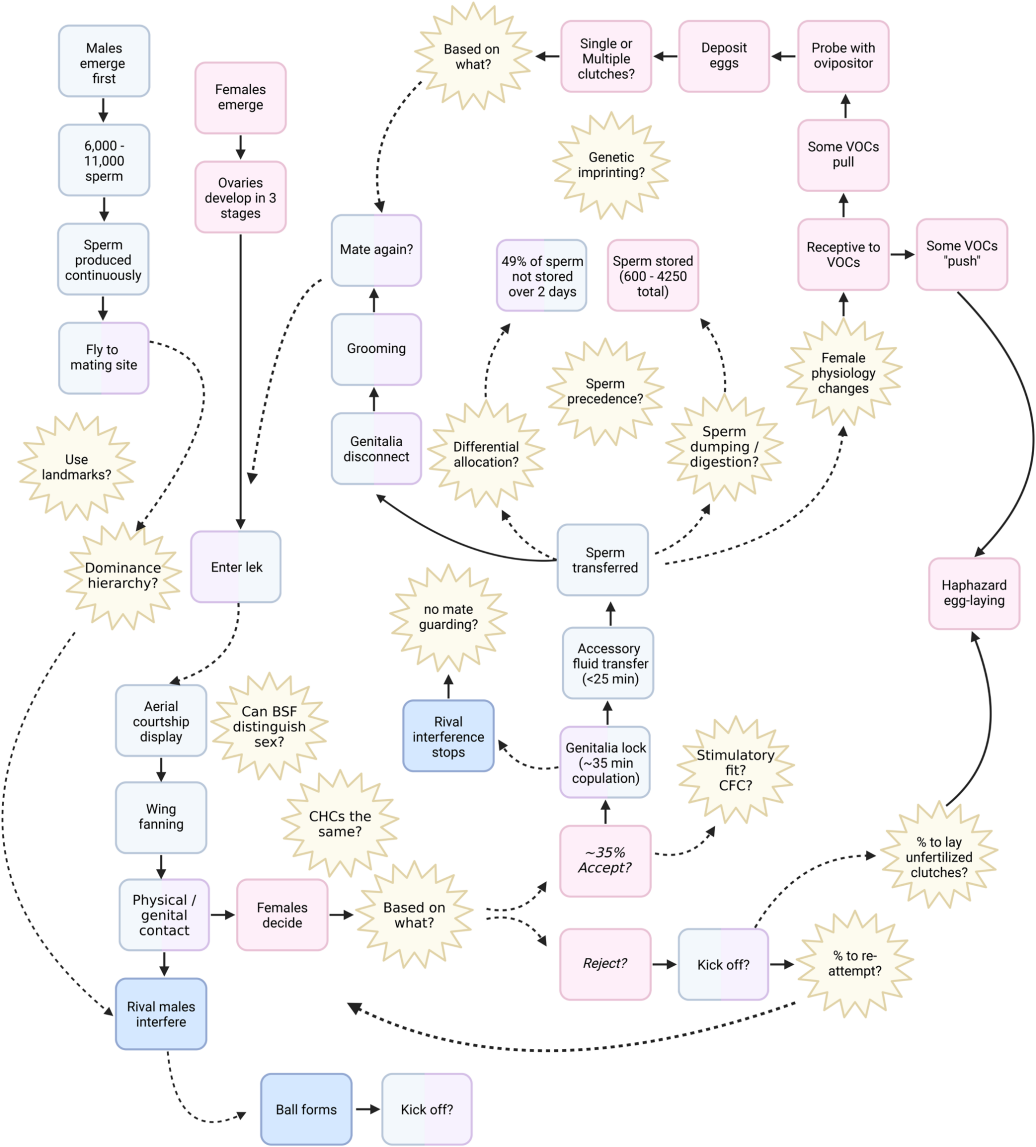
Schematic diagram depicting behavioural and physiological processes in BSF reproduction. Blue shapes represent male processes, whereas pink shapes represent female process. Shapes which are both light blue and pink are processes common to both sexes, whereas dark blue boxes are male behaviours occurring outside of the mating pair. Yellow, 12‐point stars indicate open research questions which need more exploration. Dashed arrows indicate a process is theorised but the link has yet to be demonstrated, whereas a solid arrow is one that necessarily is derived from the previous step. Created in BioRender. Lemke, N. (2026) https://BioRender.com/fy8q49x.

The previous sections highlight many important research gaps still yet to be unveiled. Here we have condensed them into a single process flow diagram depicting the BSF mating system (Figure 2), described as follows:

The onset of adult black soldier fly, *Hermetia illucens*, ontogeny begins with their protandrous emergence, where typically the earliest males emerge several days before the earliest females and establish lek‐like aggregations. However, the cues governing lek‐site selection and the establishment of dominance hierarchies remain unknown. Females subsequently visit these leks, where males initiate aerial courtship displays involving wing‐buzzing. The precise sensory modalities used in mate recognition are similarly unresolved and it is unclear whether wing‐interference patterns or acoustic signals convey species and sex identity, as are the specific traits females use to select or reject mates. Successful courtship culminates in a genital lock lasting approximately 33 min, though this duration is highly variable. The functional significance of this duration, its plasticity and the role of non‐gametic seminal fluid components in modulating female physiology are likewise yet to be determined. Following copulation, a period of intense post‐copulatory sexual selection ensues within the female's complex reproductive tract. The mechanisms governing sperm precedence and the extent to which females exert cryptic choice by differentially storing, digesting or ejecting sperm are key unanswered questions. Post reproductive females somehow then become highly responsive to volatile organic compounds that guide them to oviposition sites; but the exact mechanism for this switch in behaviour needs to be investigated. Although females use gustatory and hygroreceptors on their ovipositor to probe substrates, how they integrate these chemical cues with abiotic site characteristics to make final oviposition decisions is poorly understood. Finally, the drivers of multiple mating in both sexes as well as the timing of female remating relative to oviposition are not well defined. Disentangling the male‐ and female‐driven components of these complex pre‐ and post‐copulatory interactions are examples of untapped research opportunities in BSF reproductive biology.

## 6 Conclusions and Future Directions

1. BSF is well‐poised to be used as a research model to investigate questions in both fundamental evolutionary and applied sciences such as sustainable agriculture, waste management, biotechnology and insect welfare.
2. As a non‐pest, lekking species that is easily culturable, meaning BSF provides an avenue to study reproductive processes that are happening in a wide range of species with similar life history and reproductive traits.
3. BSF displays a capital‐income breeding hybrid strategy, allowing the study of reproductive outcomes that are mediated strictly by larval nutrition, as well as enhanced or otherwise affected by adult nutrition.
4. Future research priorities include: (i) Identifying the cues used for mate recognition and lek formation in wild and captive context; (ii) Quantifying sperm precedence patterns and female control over fertilization; (iii) Investigating the potential trade‐offs between production efficiency (larval size and nutrition), supplemental adult nutrition and breed stock resilience (adult reproduction); (iv) Documenting the variation in BSF phenome with respect to differing genetic provenances and nutritional legacies; (v) Developing management strategies to balance genetic diversity with targeted selection; and (vi) Quantifying the relative strength of pre‐ and post‐copulatory selection.
5. Achieving these will require the integration of controlled experiments, comparative fieldwork, molecular tools and machine‐learning assisted computer vision. Standardizing methodologies such as population densities, light regimes and cage designs will also improve reproducibility and comparisons across studies.

## Funding

This research was partially supported by funds from the US State Department and the Belgium‐Luxembourg Fulbright Commission under the 2026 US‐Belgium Fulbright Scholar Program. The content of this report is explicitly that of the authors and does not reflect the views of the Fulbright Program, nor the governments of the United States, Belgium, or Luxembourg. This was also partially supported by the National Research Foundation of Singapore's Intra‐CREATE Thematic Grant (NRF2020‐THE003‐0003) as well as the A*STAR Biotech in Future Foods Research Programme (R25IBIR004).

## Ethics Statement

The authors have nothing to report.

## Conflicts of Interest

The authors declare no conflicts of interest.

## Data Availability

The authors have nothing to report.
